# Effects of Plant Growth-Promoting Bacteria (PGPB) Inoculation on the Growth, Antioxidant Activity, Cu Uptake, and Bacterial Community Structure of Rape (*Brassica napus* L.) Grown in Cu-Contaminated Agricultural Soil

**DOI:** 10.3389/fmicb.2019.01455

**Published:** 2019-06-27

**Authors:** Xue-Min Ren, Shi-Jun Guo, Wei Tian, Yan Chen, Hui Han, E. Chen, Bai-Lian Li, Yu-Ying Li, Zhao-Jin Chen

**Affiliations:** ^1^Innovation Center of Water Security for Water Source Region of Mid-Route Project of South-North Water Diversion of Henan Province, School of Agricultural Engineering, Nanyang Normal University, Nanyang, China; ^2^School of Life Sciences and Technology, Nanyang Normal University, Nanyang, China; ^3^Nanjing Institute of Environmental Sciences, Ministry of Environmental Protection, Nanjing, China; ^4^Gansu Province Environmental Monitoring Centre, Lanzhou, China; ^5^Ecological Complexity and Modelling Laboratory, Department of Botany and Plant Sciences, University of California, Riverside, Riverside, CA, United States

**Keywords:** plant growth-promoting bacteria, plant-PGPB combined remediation, antioxidant activity, bacterial community, field experiment

## Abstract

Previous analyses of plant growth-promoting bacteria (PGPB) combined with the remediation of heavy metal pollution in soil have largely been performed under potting or greenhouse conditions, and *in situ* remediation experiments under field conditions have rarely been reported. In this study, the effects of the metal-resistant PGPB *Microbacterium oxydans* JYC17, *Pseudomonas thivervalensis* Y1-3-9, and *Burkholderia cepacia* J62 on soil Cu pollution under rape remediation were studied in the farmland surrounding the Nanjing Jiuhuashan copper mining region in China. Following inoculation treatment for 50 days, the biomasses of the rape inoculated with strains JYC17, Y1-3-9, and J62 increased, and the total amounts of Cu uptake increased by 113.38, 66.26, and 67.91%, respectively, the translocation factor (TF) of rape inoculated with J62 was 0.85, a significant increase of 70.68%, thus improving the Cu remediation efficiency of the rape. Y1-3-9 and J62 affected the bioavailability of Cu in the soil, and the water-soluble Cu contents were increased by 10.13 and 41.77%, respectively, compared with the control. The antioxidant activities in the rape leaves showed that the tested bacteria increased the contents of antioxidant non-enzymatic substances, including ascorbic acid (ASA) and glutathione (GSH), which were increased by 40.24–91.22% and 9.89–17.67%, respectively, thereby reducing the oxidative stress caused by heavy metals and the contents of thiobarbituric acid-reactive substances (TBARS) and peroxidase (POD). PCR-denaturing gradient gel electrophoresis (PCR-DGGE) was used to analyze the effects of the tested bacteria on the cultivation-dependent and cultivation-independent bacterial communities in the root endosphere and rhizosphere soil of the rape. The sequencing results of the DGGE bands indicated that the tested bacteria colonized the endosphere and rhizosphere, and they became an important component of the cultivation-dependent bacteria. The canonical correspondence analysis (CCA) of the DGGE profile and similarity cluster analysis showed that the tested bacteria affected the cultivation-dependent and cultivation-independent bacterial communities in the root endosphere and rhizosphere. In this experiment, the effects and mechanisms of the combined plant-microbe remediation under field conditions were preliminarily studied, and the results are expected to provide a theoretical basis for future combined remediation experiments.

## Highlights

- *In situ* combined plant-PGPB remediation experiments were conducted through field trials.
- Metal-resistant PGPB J62 increased the biomass, Cu uptake and translocated the Cu to harvestable tissues of rape.
- PGPB increased the ASA and GSH contents in the rape leaves and reduced the TBARS and POD contents.
- PGPB colonized the rape endosphere and rhizosphere and altered its bacterial community composition.

## Introduction

Due to mining and heavy metals from sewage irrigation, heavy metal-contaminated cultivated land accounts for approximately one-fifth of the total cultivated land in China. This large-scale heavy metal pollution of farmland has serious consequences for food security in China and necessitates remediation and treatment (Li et al., 2014; Zhao et al., 2015; Duan et al., 2016). Phytoremediation has become an important area of research within soil heavy metal pollution remediation because it does not produce secondary pollution, is a simple operation, and incurs low investment costs (Cunningham et al., 1995; Liu et al., 2018).

During the remediation of heavy metal-contaminated soil, plants grow slowly and have reduced biomass due to heavy metal stress, thus limiting their remediation efficiency (Ullah et al., 2015). There are many types of microorganisms in soil, which are present in large quantities, have high biological activity, and play important roles in the growth of remediation plants in heavy metal-contaminated soils and in the geochemical cycle of heavy metals (Teng et al., 2015; Chen et al., 2017; Jing and Kjellerup, 2018). Bacteria in soil can improve plant nutrition through phosphorus solubilization and nitrogen fixation and through the secretion of plant hormones [indole-3-acetic acid (IAA), etc.], siderophores, and specific enzymes [1-aminocyclopropane-1-carboxylate (ACC) deaminase, etc.], thus promoting the growth of remediation plants and the enrichment of heavy metals under stress as well as improving the heavy metal phytoremediation efficiency in soil. Due to their complementation ability, plant-microbe-combined remediation is currently a research hotspot (Glick, 2003; Ma et al., 2016; Ashraf et al., 2017). To date, researchers have inoculated hyperaccumulator plants such as *Solanum nigrum* (Luo et al., 2011), *Thlaspi caerulescens* (Whiting et al., 2001), and *Sedum plumbizincicola* (Ma et al., 2015), energy plants such as *Zea mays* (Sheng et al., 2012) and *Napier grass* (Wiangkham and Prapagdee, 2018), and high-biomass plants such as poplar (Wang et al., 2011) with plant growth-promoting bacteria (PGPB) to investigate their effects on plant growth and their heavy metal remediation efficiency in soils. However, most investigations of combined plant-PGPB remediation for soil heavy metal pollution have been performed under simple or controllable conditions such as potted plants or greenhouses. Field trials with more complicated environmental conditions have rarely been reported, while field trials are a necessary stage for combined plant-PGPB remediation technology to progress from the laboratory to practical applications, and the related work must be continued (Zhuang et al., 2007; Dary et al., 2010; Kong and Glick, 2017; Ju et al., 2019). Prapagdee and Khonsue (2015) showed that the inoculation of cadmium-resistant *Ralstonia* sp. TISTR 2219 and *Arthrobacter* sp. TISTR 2220 bacteria under field conditions increased cadmium accumulation in the roots, above-ground tissues, and whole plants of *Ocimum gratissimum*. The “*in situ*” remediation experiment conducted by Dary et al. (2010) showed that the inoculation of *Lupinus luteus* with metal-resistant PGPB increased the biomass and reduced the metal accumulation.

Rape is a cruciferous *Brassica* plant, for which many species or genotypes have strong heavy metal accumulation characteristics (Rizwan et al., 2018). Research examining the combined remediation of heavy metal pollution in soil using rape as a remediation plant inoculated with PGPB has been performed under potted or greenhouse conditions, but *in situ* field trials for related research have rarely been reported (Farwell et al., 2006; Sheng and Xia, 2006; Chen et al., 2013; Dąbrowska et al., 2017; Pan et al., 2017). Therefore, the primary objectives of this study were (1) to evaluate the effect of PGPB inoculation on the growth of rape and its enrichment of heavy metals in the field around a region characterized by heavy metal mine tailings, (2) to determine the effect of PGPB on the antioxidant activity of rape during the enrichment of soil heavy metals, and (3) to determine the effect of PGPB inoculation on the composition of the bacterial community in the rape rhizosphere and endosphere. Through the above research, we expected to perform a preliminary exploration of the PGPB mechanism that improves plant enrichment of heavy metals under field conditions from the perspectives of plant physiological indicators and microbial communities.

## Materials and Methods

### Bacterial Strain and Field Experiment Site

Three metal-resistant bacteria, *Burkholderia cepacia* J62, *Pseudomonas thivervalensis* Y1-3-9, and *Microbacterium oxydans* JYC17, which were stored in our laboratory, can produce plant growth-promoting substances such as indole-3-acetic acid (ranging from 3.3 to 10.8 mg L^–1^), siderophores, 1-aminocyclopropane-1-carboxylic deaminase (ranging from 8.0 to 307.0 μM α-KB mg^–1^ h^–1^), and solubilized inorganic phosphate (ranging from 127.0 to 234.0 mg L^–1^) (Jiang et al., 2008; He et al., 2010; Zhang et al., 2011). *B. cepacia* J62 was isolated from a Pb-contaminated paddy field in Zhejiang, China; *P. thivervalensis* Y1-3-9 was isolated from the leaves of *Mosla chinensis*; and *M. oxydans* JYC17 was isolated from the rhizosphere soils of *Kummerowia striata* grown on Cu mine wasteland. These bacterial isolates can promote plant growth and heavy metal uptake by plants from heavy metal-contaminated soils during pot experiments (Jiang et al., 2008; He et al., 2010; Zhang et al., 2011).

The field experiment site is farmland near Nanjing Jiuhuashan Cu mining, on Funiu mountain in Tangshan town (32°04’N; 119°05’E), Jiangning District of Nanjing, which was polluted by Cu mining. The soil characteristics include a pH of 6.68, organic matter content of 3.04 g kg^–1^, cation exchange capacity (CEC) of 14.5 cmol kg^–1^, total nitrogen (N) of 1.41 g kg^–1^, phosphorus (P) of 13.4 mg kg^–1^, and potassium (K) of 85.7 mg kg^–1^, along with heavy metal (Cu, Zn, Pb, and Mn) contents of 1068.25, 133.00, 34.50, and 534.75 mg kg^–1^. With reference to Soil Environmental Quality Standard Two (GB 15618-1995), the Cu pollution at the field experiment site exceeded grade III for agricultural land (400 mg kg^–1^).

### Introduction of Strains J62, JYC17, and Y1-3-9 and Plant Growth

The field experiments were performed during the season between September and November in 2012. The fields were divided into plots for rape (variety Qinyou-7) planting. The field experiments were divided into four treatments, namely, J62 inoculation, Y1-3-9 inoculation, JYC17 inoculation, and an uninoculated control. Each plot was 52 m^2^ (13 m × 4 m) in area, with plants growing in a row spacing of 0.5 m × 0.5 m (Supplementary Figure S1A). The field plot experiments were conducted using a randomized arrangement with three replications. For the inoculation, strains J62, JYC17, and Y1-3-9 were grown in LB medium for 18 h at 28°C with continuous shaking at 200 rpm, and the cells were collected by centrifugation at 6,000 rpm for 10 min, washed, and recentrifuged in sterile distilled water to obtain a bacteria inoculum of approximately 10^8^ cfu mL^–1^. The bacterial suspensions (10 mL plant^–1^) were sprayed on the rape rhizosphere. For the uninoculated control, an equal volume of sterile water was added.

Fifty days after the inoculation treatment, five plants were harvested at random from the central parts of each plot (Supplementary Figure S1B). Rhizosphere soil was obtained by first gently shaking off the loosely bound soil, while the rhizosphere soil adhering to the root system was isolated by more vigorous shaking or by hand. The above-ground tissues and roots were separated and washed extensively, first in several changes of 0.01 M EDTA and then in distilled water to remove any non-specifically bound heavy metals, and then they were dried at 80°C for 2 days before the root and above-ground tissue dry weights were determined (Supplementary Figure S1C). The root and above-ground tissues were digested in a mixture of concentrated HNO_3_ and HClO_4_ (4:1, v/v), and the Cu contents of the samples were determined with an inductively coupled-plasma optical emission spectrometer (ICP-OES) (Optima 2100 DV, Perkin Elmer). The water-soluble and NH_4_OAc-extractable Cu concentrations in the rhizosphere soils of the plants were determined by ICP-OES, and the pH of the soil (1:1 w/v water) was determined with a pH meter.

### Determination of Antioxidant Enzymes and Thiobarbituric Acid-Reactive Substances (TBARS)

For the enzyme extraction, fresh leaves (0.5 g) were homogenized in 10 mL of ice-cold potassium phosphate buffer (pH 7.0) in an ice bath by grinding using a mortar and pestle. The homogenate was centrifuged at 12,000 g for 20 min at 4°C. The supernatant was stored at 4°C and used to determine the various antioxidant enzymes. The superoxide dismutase (SOD) activity was measured through the photoreduction of nitro blue tetrazolium chloride (NBT) (Dhindsa and Matowe, 1981). The peroxidase (POD) activity was measured according to the method by Rao et al. (1996). The glutathione (GSH) was measured according to Griffith (1980), and the ascorbic acid (ASA) was measured according to Arakawa et al. (1981). The protein content was determined according to the Bradford (1976) method with bovine serum albumin as the standard. The TBARS was measured as described by Jiang and Zhang (2001).

### DNA Extracted From Biomass Was Accumulated on an Agar Plate (Cultivation-Dependent) and Extracted Directly From the Endosphere and Rhizosphere Samples (Cultivation-Independent)

Culturable endophytic and rhizosphere bacteria were isolated by traditional plate culture methods. The plant roots were surface-sterilized by sequential immersion in 75% (v/v) ethanol for 2 min and 1% mercuric chloride for 1 min, they were ground with a mortar and pestle in the presence of 5 ml of sterile distilled water, and then they were spread on plates containing 1/5-strength LB medium for 72 h at 28°C to isolate the endophytic bacteria. Samples of rhizosphere soil weighing 1 g were taken from each treatment, homogenized in 10 mL of 0.85% saline, and serially diluted (10-fold) in the same container, and the aliquots (100 μL) were spread on 1/5 LB medium and incubated for 72 h at 28°C. DNA was extracted from the bacterial biomass on the plates by cetyltrimethylammonium bromide (CTAB) method (Ellis et al., 2003), and the results were considered “cultivation-dependent” samples.

Endophytic bacteria from the plant roots was extracted as described by Sun et al. (2010). The plant roots were surface-sterilized as described above for the isolation of culturable endophytic bacteria, they were ground in a sterilized mortar with 5 mL sodium phosphate buffer, and then they were transferred to tubes that were shaken for 1 h to dislodge the bacterial cells from inside the plant tissue. The bacterial cells were collected by centrifugation at 12000 g for 10 min and resuspended in 550 μL of TE buffer. The total endophytic bacterial DNA was extracted as described by Araújo et al. (2002). The rhizosphere bacterial DNA was extracted based on a modification of a method by Zhou et al. (1996). The resulting extracts were considered “endosphere” and “rhizosphere” samples.

### PCR-Denaturing Gradient Gel Electrophoresis (DGGE) Analyses

The DGGE primers GC-341F and 534R were used to amplify the V3 hypervariable region of 16S rRNA genes directly from the DNA samples (Muyzer et al., 1993). The PCRs were performed in a PTC-200 DNA Engine Cycler (Bio-Rad, United States) using the amplification program and reaction conditions described by Chen et al. (2013). For the DGGE analysis, the PCR products generated from each sample were separated on an 8% acrylamide gel with a linear denaturant gradient ranging from 45 to 75% using a DCode^TM^ Universal Mutation Detection System from BIO-RAD, United States. The DGGE was performed using 20 μL of the PCR product in 1× TAE buffer at 60°C, 200 V for 10 min, then 85 V for 12 h. The gels were stained with SYBR Green I (Generay Biotech Co., Ltd., Shanghai, China) in 1× TAE for 30 min, and the gels were scanned with a gel photo GS-800 system (Bio-Rad, United States).

The bands of interest were excised and eluted with 50 μL of ddH_2_O for 24 h at 4°C. The resulting solution (1 μL) was used as the target DNA for a subsequent PCR amplification with primers 341F and 534R, and the PCR products were cloned into the pGEM-T Easy vector as described by the manufacturer. The clones were sequenced, and these short fragments were subjected to BLAST-assisted searches of the NCBI database; the closest match of known phylogenetic affiliation was used to assign the bands to taxonomic groups.

### Statistical and Cluster Analyses

The microbial community banding profiles on the DGGE gels were analyzed using a Quantity One software package (Bio-Rad Laboratories, Inc., Hercules, United States). The plant growth parameter data were analyzed using a Student–Newman–Keuls test at a significance level of *P* < 0.05. All the statistics were performed using SPSS 13.0 for Windows (SPSS Inc., Chicago, IL, United States).

## Results

### Effects of Each Strain on the Biomass and Cu Enrichment in Rape

**FIGURE 1:**
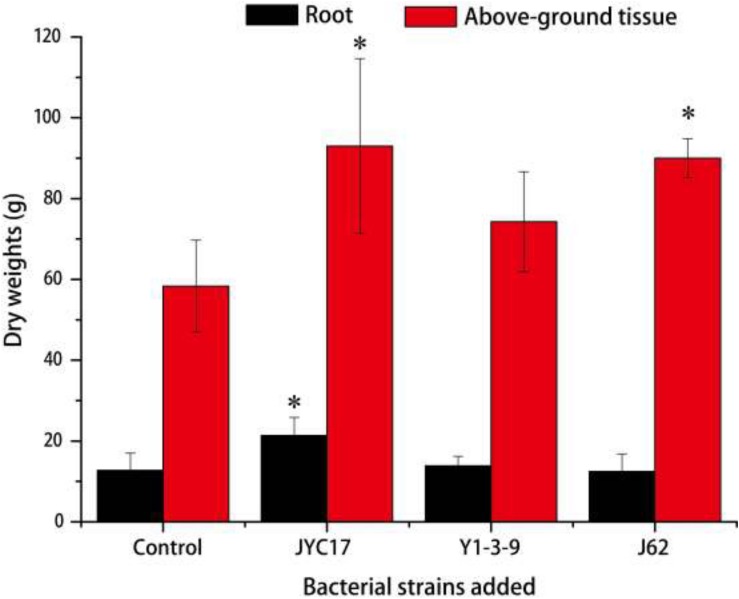
The dry weights of plants that were uninoculated and inoculated with treatments. The error bars are ± the standard deviation. An asterisk (^*^) denotes a significantly different value between the uninoculated and inoculated treatments (*P* < 0.05).

**FIGURE 2:**
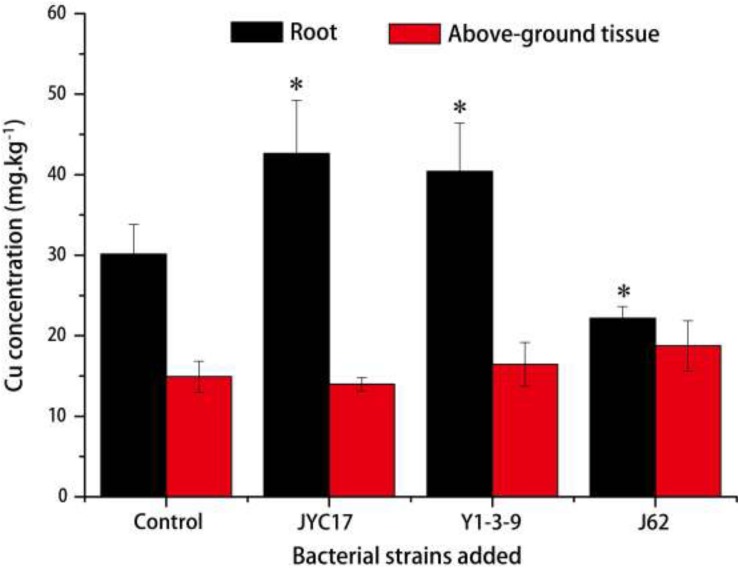
The Cu concentrations in uninoculated and inoculated plant treatments. The error bars are ± the standard deviation. An asterisk (^*^) denotes a significantly different value between the uninoculated and inoculated treatments (*P* < 0.05).

**FIGURE 3:**
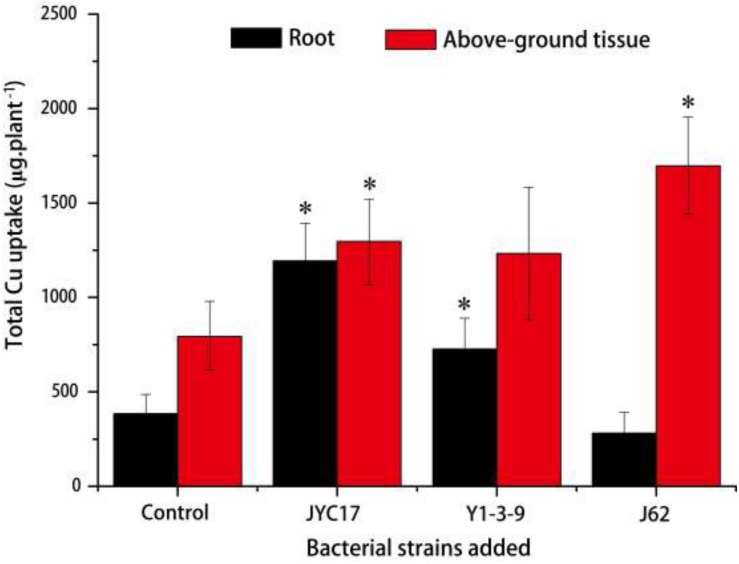
The total Cu uptake by plants in the uninoculated and inoculated treatments. The error bars are ± the standard deviation. An asterisk (^*^) denotes a significantly different value between the uninoculated and inoculated treatments (*P* < 0.05).

The remediation rape plants grew well in the Cu-contaminated farmland, and the inoculation treatment promoted plant growth. The promotion of above-ground biomass (dry weight) was more obvious than the promotion of root growth. Compared with the plants without inoculation treatment, the above-ground biomass after the inoculation treatment increased by 27.3–59.5%, and the root biomass increased by 8.6–67.2%. The increases in the above-ground biomass of rape inoculated with strains JYC17 and J62 were significantly different from those for the control without inoculation treatment (*P <* 0.05) (Figure 1). Simultaneously, the inoculation treatment affected the Cu concentration in the rape to varying degrees. The JYC17 and Y1-3-9 increased the Cu concentration in the roots of the rape, with values of 42.63 and 40.14 mg kg^–1^, respectively, demonstrating increases of 41.48 and 34.13%, compared with 30.13 mg kg^–1^ in the control group, for which the former difference was statistically significant (*P <* 0.05). Y1-3-9 and J62 increased the Cu concentrations in the above-ground part of the rape by 10.27 and 25.56%, respectively (Figure 2). The translocation factor (TF) indicates the capacity of the plants to translocate heavy metals from the roots to the above-ground tissues, which is expressed as the ratio of the heavy metal concentration in the above-ground tissues/the heavy metal concentration in the roots. The experimental results showed that the TFs of plants inoculated with JYC17, Y1-3-9, and J62 were 0.33, 0.41, and 0.85, respectively. Strain J62 significantly increased the TF by 70.68% compared with the control (*P* < 0.05). Although the tested bacteria showed different effects on the Cu concentration in different parts of the rape, the inoculation treatment increased the above-ground biomass of the plant, such that the total amount of Cu enrichment in the rape showed different degrees of improvement compared with the control. The total amounts of Cu enrichment in the roots of each rape plant inoculated with JYC17 and Y1-3-9 were 1193.90 and 727.02 μg plant^–1^, respectively, demonstrating 210.2 and 88.9% significantly higher enrichment compared with the control (*P <* 0.05). The total amounts of Cu enrichment in the above-ground part of each rape plant inoculated with JYC17, Y1-3-9, and J62 were 1296.87, 1232.14, and 1696.69 μg plant^–1^, respectively, demonstrating increases of 63.4, 55.3, and 113.8% compared with the control value of 793.47 μg plant^–1^. The total Cu enrichment in whole plants inoculated with JYC17, Y1-3-9, and J62 increased by 113.4, 66.3, and 67.9%, respectively (Figure 3).

### Effect of Strain Inoculation on the Soil Bioavailability of Heavy Metals

The bioavailability of heavy metals in the plant rhizosphere is an important factor limiting the efficiency of phytoremediation. The effects of different treatments on the forms of Cu in the rape rhizosphere soil was determined, and the results are shown in Table 1. Accordingly, the inoculation using the tested bacteria changed the forms of Cu in the soil. Strains Y1-3-9 and J62 increased the levels of water-soluble Cu in the rape rhizosphere soil to 1.12 and 0.87 mg kg^–1^, respectively, demonstrating increases of 41.77 and 10.13% compared with the control. The contents of NH_4_OAc-extracted and DTPA-extracted Cu after the inoculation treatment were lower than those in the control group, to different degrees. The inoculation showed little effect on the pH of the rhizosphere soil.

**TABLE 1:** The influence of the test strains on the number of cultivation-dependent bacteria in the rape rhizosphere and endosphere, the water-soluble Cu, NH_4_OAc-extractable Cu, DTPA-extracted Cu, and the pH in the rhizosphere soil.

**Treatments**	**Number of endophytic bacteria (cfu g^–1^ fresh weight)**	**Number of rhizosphere bacteria (cfu g^–1^ soil)**	**Water-soluble Cu (mg kg^–1^)**	**NH_4_OAc-extracted Cu (mg kg^–1^)**	**DTPA-extracted Cu (mg kg^–1^)**	**pH**
Control	2.86 × 10^4^	1.64 × 10^6^	0.79 ± 0.08^b^	13.7 ± 1.77^a^	297.2 ± 12.7^a^	7.82
JYC17	4.56 × 10^4^	3.08 × 10^6^	0.54 ± 0.07^c^	10.8 ± 3.51^abc^	243.3 ± 17.9^b^	7.84
Y1-3-9	4.45 × 10^4^	1.70 × 10^6^	1.12 ± 0.19^a^	8.98 ± 0.95^c^	249.6 ± 37.6^b^	7.91
J62	1.64 × 10^4^	2.62 × 10^6^	0.87 ± 0.23^b^	11.2 ± 1.20^b^	228.1 ± 9.17^b^	8.13

*Means within the same column followed by the same letter are not significantly different at *P* < 0.05, based on the Student–Newman–Keuls test.*

### The Effect of Strain Inoculation on the Antioxidant Activities in Rape Leaves

**FIGURE 4:**
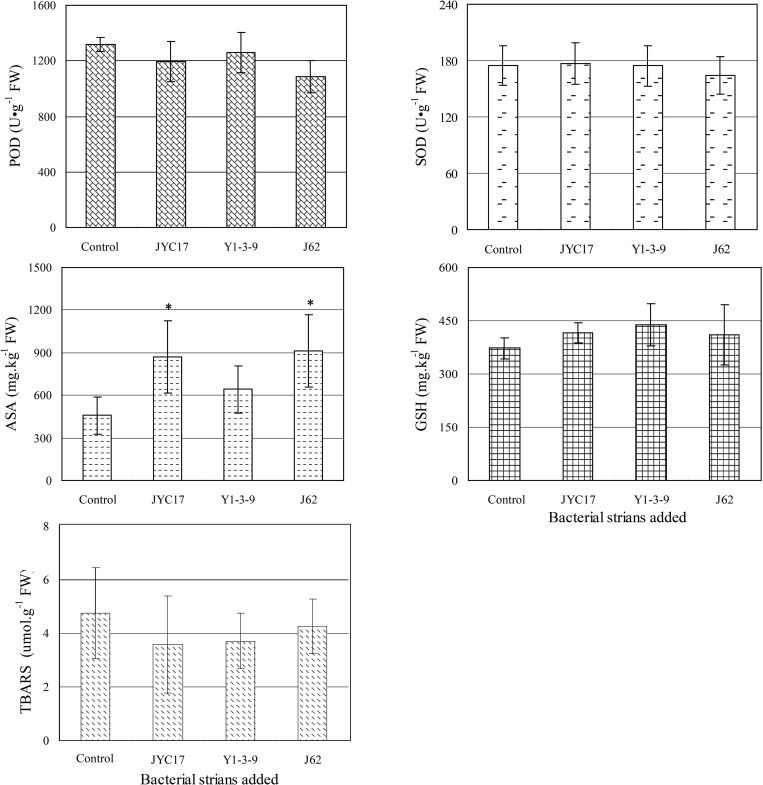
Effect of PGPB inoculation on the leaf POD, SOD, ASA, GSH, and TBARS contents of rape under metal stress. An asterisk (^*^) denotes a significantly different value between uninoculated and inoculated treatments (*P* < 0.05).

Under an environment of heavy metal stress, non-enzymatic substances composed of ASA and GSH can facilitate a plant’s elimination of peroxidation. According to the ASA content measurements shown in Figure 4, the inoculation using the tested bacteria increased the ASA contents in the rape leaves. The ASA content increased from 456.68 mg kg^–1^ fresh weight (FW) in the control to 873.26 mg kg^–1^ FW in the plants inoculated with JYC17 (91.22% increase), 640.47 mg kg^–1^ FW in the plants inoculated with Y1-3-9 (40.24% increase), and 913.37 mg kg^–1^ FW in the plants inoculated with J62 (80.49% increase). Similar to the ASA results, inoculation using the tested bacteria increased the GSH contents in the rape leaves. The GSH content increased from 373.47 mg kg^–1^ FW in the control to 415.15 mg kg^–1^ FW in the plants inoculated with JYC17 (11.16% increase), 439.46 mg kg^–1^ FW in the plants inoculated with Y1-3-9 (17.67% increase), and 410.41 mg kg^–1^ FW in the plants inoculated with J62 (9.89% increase).

The TBARS content in plants is a physiological indicator of plant stress. The increase in the TBARS content in a plant indicates that the plant is under a certain degree of stress. The TBARS contents in rape leaves inoculated with JYC17, Y1-3-9, and J62 were decreased to different extents compared with the control (10.16, 10.58, and 21.88%, respectively). Similar to the effect of TBARS in plants, the SOD and POD activities in plants exposed to adverse conditions such as heavy metal stress can be enhanced, and the reactive oxygen species (ROS) caused by the stress in the plants can be scavenged. Therefore, they can also be used as a physiological indicator of plant stress. According to the SOD and POD activity assay shown in Figure 4, JYC17, Y1-3-9, and J62 inoculation reduced the POD activity in the rape leaves by 9.39, 4.55, and 17.80%, respectively. Concomitantly, inoculating with strain J62 reduced the SOD activity in the rape leaves by 5.99%; the remaining two groups showed little change compared with the control. Combined with the changing TBARS pattern in the plants, the results suggested that inoculating with the tested bacteria reduced the physiological indicator of plant stress in the Cu-polluted farmland soil for rape.

### Composition of the Cultivation-Dependent and Cultivation-Independent Bacterial Community on the Rape Root Endosphere and Rhizosphere Soil

**FIGURE 5:**
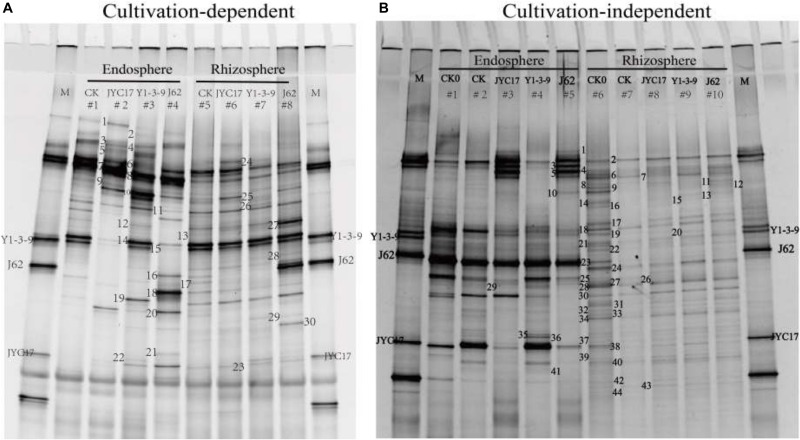
DGGE gels showing diverse 16S rRNA gene fragments amplified from panel **(A)** DNA extracted from biomass accumulated on an agar plate (cultivation-dependent) and panel **(B)** DNA extracted directly from endosphere and rhizosphere samples (cultivation-independent). **(A)** Lanes 1 to 4, endosphere samples from the 50-day uninoculated control (E-CK), 50-day inoculation with JYC17 (E-JYC17), 50-day inoculation with Y1-3-9 (E-Y1-3-9), and 50-day inoculation with J62 (E-J62); lanes 5 to 8, rhizosphere samples from the 50-day uninoculated control (R-CK), the 50-day inoculation with JYC17 (R-JYC17), the 50-day inoculation with Y1-3-9 (R-Y1-3-9), and the 50-day inoculation with J62 (R-J62). **(B)** Lanes 1 to 5, endosphere samples from the 0-day uninoculated control (E-CK0), the 50-day uninoculated control (E-CK), the 50-day inoculation with JYC17 (E-JYC17), the 50-day inoculation with Y1-3-9 (E-Y1-3-9), and the 50-day inoculation with J62 (E-J62); lanes 6 to 10, rhizoplane samples from the 0-day uninoculated control (R-CK1), the 50-day uninoculated control (R-CK2), the 50-day inoculation with JYC17 (R-JYC17), the 50-day inoculation with Y1-3-9 (R-Y1-3-9), and the 50-day inoculation with J62 (R-J62). M, marker lane consisting of 16S rRNA gene fragments from J62, JYC17 and Y1-3-9. The bands in the DGGE profiles were excised and sequenced, and their given numbers correspond to the list in Supplementary Table S1.

The band mobility of each sample in the DGGE profile was analyzed using Quantity One analysis software. A total of 23 bands from the cultivation-dependent profile and 43 bands from the culture-independent profile were cut and recovered (Figure 5). These bands were sequenced, and a homology alignment was performed with the known sequences in GenBank using BLAST software. The application of accession number HQ603005-HQ603051 was submitted to the GenBank database. Based on the sequencing results for the bands, the composition of the cultivation-dependent bacterial community in the rape root endosphere and its rhizosphere soil in the heavy metal-contaminated farmland near the Cu mine was as follows: Gammaproteobacteria, Firmicutes, Betaproteobacteria, and Actinobacteria, with 10, 7, 3, and 1 bands and proportions of the 21 sequenced bands at 47.6, 33.3, 14.3, and 4.7%, respectively (Figure 5A and Supplementary Table S1). *Bacillus* spp. in Gammaproteobacteria and *Pseudomonas* spp. in Firmicutes were the dominant populations among the cultivation-dependent bacteria, accounting for 47.6 and 28.6% of the total numbers of sequenced bands, respectively. The bands in the cultivation-independent DGGE profile of the directly extracted DNA samples were sequenced, and they primarily consisted of seven bacterial populations, including Alphaproteobacteria (1 band, 3.8%), Betaproteobacteria (2 bands, 7.7%), Gammaproteobacteria (5 bands, 19.2%), Firmicutes (2 bands, 7.7%), Actinobacteria (2 bands, 7.7%), Bacteroidetes (3 bands, 11.5%), and uncultured bacteria (11 bands, 42.3%) (Figure 5B and Supplementary Table S1). *Rhodanobacter* spp. and *Pseudomonas* spp. in Gammaproteobacteria and *Burkholderia* sp., *Janthinobacterium* sp. and *Achromobacter* sp. in Bacteroidetes were the dominant populations in the endosphere and rhizosphere samples.

### Effects of the Tested Bacteria on the Bacterial Communities in the Endosphere and Rhizosphere

The effects of the tested bacteria on the number of cultivation-dependent bacteria in the rape rhizosphere and endosphere were determined. The results showed that the total number of bacteria in the rhizosphere of the tested plants was 10^6^ cfu g^–1^ soil and that the total number of bacteria in the roots was approximately 10^4^ cfu g^–1^ fresh weight. Inoculating with Y1-3-9 and JYC17 increased the number of bacteria in the rhizosphere and the roots. Simultaneously, the sequencing results for the DGGE bands showed that the sequence information for bands A15, A16, and A23 corresponded to the test bacteria Y1-3-9, J62, and JYC17, respectively, with a similarity of 100%. Based on the DGGE sequencing results and the counts of cultivation-dependent bacteria, the tested bacteria colonized the rape endosphere and rhizosphere under field conditions (Table 1, Supplementary Table S1, and Figure 5A).

**FIGURE 6:**
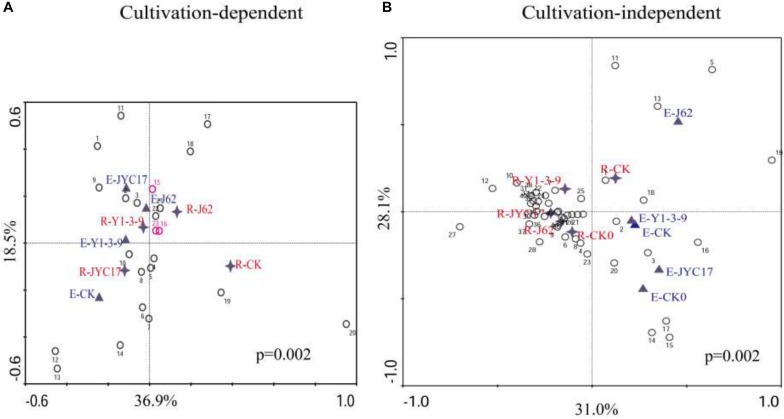
Canonical correspondence analysis (CCA) of DGGE bacterial community profiles from panel **(A)** DNA extracted from biomass accumulated on an agar plate (cultivation-dependent, Figure 5A) and panel **(B)** DNA extracted directly from endosphere and rhizosphere samples (cultivation-independent, Figure 5B). The values on the axes indicate the total variation percentages explained by each axis. **(A)** Endosphere samples from the 50-day uninoculated control (E-CK), 50-day inoculation with JYC17 (E-JYC17), 50-day inoculation with Y1-3-9 (E-Y1-3-9), and 50-day inoculation with J62 (E-J62); rhizosphere samples from the 50-day uninoculated control (R-CK), the 50-day inoculation with JYC17 (R-JYC17), the 50-day inoculation with Y1-3-9 (R-Y1-3-9), and the 50-day inoculation with J62 (R-J62). The digital numbers represent 23 bands from the cultivation-dependent profile (Figure 5A). **(B)** Endosphere samples from the 0-day uninoculated control (E-CK0), the 50-day uninoculated control (E-CK), the 50-day inoculation with JYC17 (E-JYC17), the 50-day inoculation with Y1-3-9 (E-Y1-3-9), and the 50-day inoculation with J62 (E-J62); rhizoplane samples from the 0-day uninoculated control (R-CK1), the 50-day uninoculated control (R-CK2), the 50-day inoculation with JYC17 (R-JYC17), the 50-day inoculation with Y1-3-9 (R-Y1-3-9), and the 50-day inoculation with J62 (R-J62). The digital numbers represent 43 bands from the culture-independent profile (Figure 5B).

To investigate the effects of the tested bacteria on the bacterial community structure of the rape endosphere and its rhizosphere, the DGGE profile was subjected to a canonical correspondence analysis (CCA), and the results are shown in Figure 6. Figure 6A shows the CCA of the cultivation-dependent DGGE profile. The cultivation-dependent bacterial community in the endosphere (E-CK) and the cultivation-dependent bacterial community in the rhizosphere (R-CK) in the control group without strain inoculation was separated through axis 1 (36.9%), indicating that the structure of the cultivation-dependent bacterial communities in the root endosphere was significantly different from that of the cultivation-dependent bacterial communities in the rhizosphere soil. Following inoculation with the tested bacteria, the endosphere cultivation-dependent bacteria community and rhizosphere cultivation-dependent bacteria community were clustered together above axis 2 (18.5%) of the CCA profile, in which the endosphere cultivation-dependent bacteria (E-J62) and rhizosphere cultivation-dependent bacteria (R-J62) for strain J62 and the endosphere cultivation-dependent bacteria (E-Y1-3-9) and rhizosphere cultivation-dependent bacteria (R-Y1-3-9) for strain Y1-3-9 were clustered together and separated from those of the control group (E-CK and R-CK). This finding indicates that the test bacteria J62 and Y1-3-9 affected the structure of the cultivation-dependent bacterial communities in the rape endosphere and its rhizosphere and had similar community structures.

Figure 6B shows the CCA of the cultivation-independent DGGE profile. Axis 1 divided the endosphere bacteria and rhizosphere bacteria into two parts at 31.0%, indicating that different sample sources (the rape endosphere and rhizosphere) are the most important factors determining the structure of the bacterial communities. Based on the CCA, the community structure in the control group at day 0 for the endosphere bacteria (E-CK0) and rhizosphere bacteria (R-CK0) was separated from the community structure at day 50 for the endosphere bacteria (E-CK) and rhizosphere bacteria (R-CK), indicating that the bacterial community changed over time. The E-Y1-3-9, E-JYC17, and E-J62 for the rape endosphere following inoculation were separated from each other in the CCA profile, while E-Y1-3-9 was closer to the E-CK of the control group, indicating that inoculation with the tested bacteria affected the bacterial communities of the rape root endosphere and that the degrees of influence from the different strains were different. Similarly, the R-Y1-3-9, R-JYC17, and R-J62 for the rhizosphere after inoculation treatment were clustered together in the CCA profile and separated from the R-CK of the control group, indicating that the inoculation with the tested bacteria affected the bacterial communities of the rape rhizosphere soil.

A bacterial community similarity clustering analysis was performed on the DGGE profile according to the unweighted pair group method with the arithmetic mean (UPGMA) algorithm, which was similar to the CCA results. The cultivation-dependent bacteria in the endosphere samples were clustered at a similarity level of 0.39 and were separated from the rhizosphere samples, indicating that the different sample source was the most important factor affecting the composition of the cultivation-dependent bacterial communities (Supplementary Figure S2). Concurrently, the inoculated and non-inoculated samples were separated from each other in the cluster analysis profile, indicating that inoculating with the tested bacteria affected the composition of the cultivation-dependent bacterial communities in the rape endosphere and rhizosphere. Similarly, the cultivation-independent bacteria in the rhizosphere samples were clustered at a similarity level of 0.43 and were separated from the rhizosphere samples. Simultaneously, the inoculated and non-inoculated samples were separated from one another in the cluster analysis profile.

## Discussion

During the combined plant-microbe remediation of heavy metal pollution in soil, PGPB can regulate the physiological processes of plants and reduce the stress from heavy metals on plants; simultaneously, the strains can activate insoluble heavy metals and phosphorus nutrients in the soil by producing iron carriers and organic acids. These bacteria can change the bacterial community structure of the rhizosphere soil and its endosphere and improve the biomass of the remediation plant, thus improving the efficiency of phytoremediation (Teng et al., 2015; Ma et al., 2016; Ashraf et al., 2017). However, microbes have a strong dependence on the environment, and changes in environmental conditions can modulate the effects of microbes. To date, studies related to the plant-PGPB remediation of heavy metal pollution in soil have primarily been performed under conditions that are relatively easy to control, such as in pots or in a greenhouse. Few studies have examined the effects of these strains under complex field conditions, limiting the practical application of combined plant-PGPB remediation (Zhuang et al., 2007; Dary et al., 2010; Kong and Glick, 2017). The remediation field trials conducted by Prapagdee and Khonsue (2015) and Dary et al. (2010) showed that the inoculation of metal-resistant PGPB improved the biomass of the remediation plant *L. luteus* and achieved the purpose of increasing or decreasing the metal accumulation in *O. gratissimum* and *L. luteus*. The experimental results showed that the combined plant-PGPB remediation of heavy metal pollution in soil has a certain practical value under field conditions. In our previous pot trials, metal-resistant PGPB J62 and Y1-3-9 reduced the Cd stress in rape and increased the total amount of Cd uptake by the plant. The cumulative amount was increased from 6.70 to 40.60% compared with the corresponding control (Chen et al., 2013). The results of the combined remediation field trials showed that inoculating with the tested bacteria promoted rape growth, increased the dry weight of the above-ground tissues and roots, affected the Cu concentration in the rape and increased the total amount of Cu uptake. The total Cu uptake in whole plants inoculated with JYC17, Y1-3-9, and J62 was increased by 113.38, 66.26, and 67.91%, respectively. PGPB can directly or indirectly dissolve insoluble heavy metals through various metabolic activities (Sessitsch et al., 2013). Strains Y1-3-9 and J62 can activate water-soluble Cd in the soil in pot experiments (Chen et al., 2013). Similarly, strains Y1-3-9 and J62 can increase the contents of water-soluble Cu in rape rhizosphere soil under field conditions. The above results indicate that strains JYC17, Y1-3-9, and J62 are effective at promoting the phytoremediation of heavy metal pollution in soil under field conditions and that the rape remediation system and strains Y1-3-9 and J62 have good adaptability, which can be applied to different conditions (potted plants and fields) for different heavy metal types (Cu and Cd pollution), showing a certain application value. However, the current experiments were conducted only for a single rape crop in a single soil contaminated with heavy metals. Experimental research to account for different crops, soil types, and growing environments is needed to investigate the combined remediation of heavy metal pollution in the soil using strains JYC17, Y1-3-9, and J62 under field conditions.

Heavy metals in the soil often cause direct or indirect damage, inhibit the growth of remediation plants, and affect the efficiency of phytoremediation. Studies have shown that PGPB can alleviate oxidative stress and reduced chlorophyll synthesis, impact other physiological and biochemical indicators caused by heavy metals, and improve the adaptability of remediation plants to heavy metal pollution (Tak, 2015; Pan et al., 2016; Chiboub et al., 2018; Rizvi and Khan, 2018). Saleem et al. (2018) found that Pb-tolerant PGPB increased the biomass and Pb uptake of sunflower remediation plants in pot experiments. Simultaneously, the indicators of physiological and antioxidant activities showed that PGPB increased the contents of chlorophyll “a,” chlorophyll “b,” carotenoids, ascorbate peroxidase, catalase, superoxide dismutase, glutathione reductase, and proline in sunflowers that were exposed to Pb contamination. These indicators play an important role in improving the photosynthetic activities and alleviating the oxidative stress caused by heavy metals. Similarly, this experiment determined the effects of inoculating the rape with PGPB on the antioxidant indicators under field conditions. The results showed that PGPB inoculation increased the contents of the non-enzymatic antioxidants ASA and GSH by 40.24–91.22% and 9.89–17.67%, respectively. Studies have shown that ASA and GSH are the most important antioxidants in plants, and they play important roles in scavenging the toxic ROS caused by heavy metal stress. The increased contents of these compounds help plants to tolerate various stresses. This result is similar to the findings of Pan et al. (2016) and Islam et al. (2014), who showed that inoculation by the tested bacteria increased the GSH and ASA contents and alleviated the oxidative stress caused by heavy metals. The TBARS, SOD, and POD contents in plants reflect the changes in ROS and hydrogen peroxide free radicals in plant tissues, so they can be used as indicative physiological indicators of plant stress. The TBARS and POD contents in the rape leaves treated with JYC17, Y1-3-9, and J62 were decreased to different degrees compared with the control, indicating that the oxidative stress caused by heavy metals was alleviated in the rape after the inoculation treatment.

Microorganisms are widely distributed in the plant rhizosphere and endosphere, and they play important roles in the phytoremediation of heavy metal-contaminated soil (Ashraf et al., 2017). PCR-DGGE is a modern molecular biology technology for studying microbial communities. It is widely used in microbial ecology investigations in different ecological environments such as plant samples (de Melo Pereira et al., 2012; Chen et al., 2013) and soil (Che et al., 2015; Orlewska et al., 2018). Rhizosphere and endosphere colonizations of PGPB in plants are the primary conditions for its effect. Because of its technical sensitivity, PCR-DGGE technology has been used to study strain colonization in soils or plants (Andreote et al., 2009; Chen et al., 2013; Rilling et al., 2019). Using the PCR products of strains JYC17, Y1-3-9, and J62 as the DGGE markers, and based on the positions of the corresponding bands on the cultivation-dependent and cultivation-independent DGGE profiles and the sequencing information for bands A15, A16, and A23 (Supplementary Table S1 and Figure 5), it was judged that the tested bacteria colonized the rape roots and rhizosphere under field conditions, consistent with the colonization results obtained for strains J62 and Y1-3-9 in the rape rhizosphere and endosphere during previous potting trials (Chen et al., 2013). Studies concerning the interaction of PGPB with microorganisms in soil or the plant endosphere during combined plant-microbe remediation, as well as the impact on the composition of the microbial community in soil, have also been conducted (Jeong et al., 2013; Marques et al., 2013; Liu et al., 2015). Liu et al. (2015) showed that the inoculation of heavy metal-tolerant PGPB during the remediation of Cd-contaminated soil by *S. plumbizincicola* reduced the diversity of the rhizosphere microbial community. Cultivation-dependent bacteria play an important role in the microbial community and its activity. In traditional studies that have examined the composition of cultivation-dependent bacterial communities, cultivation-dependent bacteria are generally first enriched and cultured in a medium, followed by separation, purification, and identification, which is time-consuming and laborious. Based on the results reported by Ellis et al. (2003), the total DNA from bacteria growing on a plate were extracted, and a PCR-DGGE analysis was performed to analyze the effects of the tested bacteria quickly and accurately on the composition of the microbial community of the cultivation-dependent bacteria in the rape rhizosphere soil and root endosphere (Supplementary Table S1 and Figure 5). Using CCA and similarity clustering analyses of the cultivation-dependent DGGE profile, it was concluded that the tested bacteria inoculated with JYC17, Y1-3-9, and J62 under field conditions affected the composition of the cultivation-dependent bacterial communities in the rape root endosphere and rhizosphere soil and that the effects of the different strains were different. These results were consistent with previous findings demonstrating that the cultivation-dependent bacterial community composition of the rape rhizosphere and endosphere was affected by strains J62 and Y1-3-9 under potting conditions (Chen et al., 2013). Similar to the change in the cultivation-dependent bacterial community composition, the cultivation-independent DGGE profile results showed a significant difference between the rape root and rhizosphere. Inoculation with the test bacteria affected the composition of the bacterial communities in the endosphere and rhizosphere to different extents. These results are similar to previous findings showing that inoculation by plant growth-promoting rhizobacteria can alter the bacterial community composition of the plant rhizosphere or endosphere (Andreote et al., 2010; Chen et al., 2013; Liu et al., 2015). These changes in the community composition, especially the colonization of the test bacteria JYC17, Y1-3-9, and J62 with good growth-promoting abilities in the rape roots and rhizosphere, significantly enhanced the proportion of growth-promoting bacteria among the total cultivation-dependent bacteria, which may play an important role in the heavy metal stress tolerance of rape.

## Conclusion

The results of the *in situ* remediation experiments on rape-promoting bacteria in the Cu-contaminated farmland around heavy metal mine tailings showed that metal-resistant PGPB increased the rape biomass and the total amount of Cu uptake, thus enhancing the Cu enrichment efficiency of rape. Simultaneously, the mechanism of this effect was preliminarily analyzed from the perspectives of the plant physiology and the microbial community structure. The results showed that the test bacteria JYC17, Y1-3-9, and J62 had good growth-promoting abilities, colonized the rape rhizosphere and endosphere, and altered the bacterial community composition of the rape rhizosphere and endosphere. Concurrently, they increased the ASA and GSH contents in the rape and reduced the oxidative stress caused by heavy metals. The above results indicate that the plant-promoting bacteria JYC17, Y1-3-9, and J62 are effective at promoting the phytoremediation of heavy metal pollution in soil under field conditions, and they could have value for use in specific applications.

## Data Availability

The raw data supporting the conclusions of this manuscript will be made available by the authors, without undue reservation, to any qualified researcher.

## Author Contributions

Z-JC designed the experiments and contributed to writing the manuscript. X-MR and S-JG performed the experiments and analyzed the data. HH and YC performed the field sampling. WT and EC measured the heavy metals concentrations in the rape. B-LL and Y-YL reviewed the manuscript.

## Conflict of Interest Statement

The authors declare that the research was conducted in the absence of any commercial or financial relationships that could be construed as a potential conflict of interest.
