# Supplementary material for: Effects of Plant Growth-Promoting Bacteria (PGPB) Inoculation on the Growth, Antioxidant Activity, Cu Uptake, and Bacterial Community Structure of Rape (Brassica napus L.) Grown in Cu-Contaminated Agricultural Soil
Source: Front Microbiol. 2019 Jun 27;10:1455. doi: 10.3389/fmicb.2019.01455 (PMC6610483; doi:10.3389/fmicb.2019.01455)
Supplement: Supplementary file 1 [file Table_1.doc]

**Effects of plant growth-promoting bacteria (PGPB) inoculation on the growth, antioxidant activity, Cu uptake, and bacterial community structure of rape (*Brassica napus* L.) grown in Cu-contaminated agricultural soil**

Xue-Min Ren ^a^**^†^**, Shi-Jun Guo ^b^**^†^**, Wei Tian ^c^, Yan Chen ^b^, Hui Han ^a^, E Chen ^d^, Bai-Lian Li ^a,e^, Yu-Ying Li ^a^, Zhao-Jin Chen ^a,*^

^a^ Innovation Center of Water Security for Water Source Region of Mid-route Project of South-North Water Diversion of Henan Province, School of Agricultural Engineering, Nanyang Normal University, Nanyang 473061, China

^b^ School of Life Science and Technology, Nanyang Normal University, Nanyang 473061, China

^c^ Nanjing Institute of Environmental Sciences, Ministry of Environmental Protection, Nanjing 210042, China

^d^ Gansu Province Environmental Monitoring Centre, Lanzhou 730020, China

^e^ Ecological Complexity and Modelling Laboratory, Department of Botany and Plant Sciences, University of California, Riverside, CA 92521, USA

^†^ These authors contributed equally to this work.

^*^ Corresponding author.

E-mail: Zhaojin_chen@163.com (Z. Chen)

Figure S1. The field experiments were divided into plots for rape planting (A). Rape were inoculated with strains J62, JYC17, and Y1-3-9, harvested at random from the central parts of each plot (B, C).

Figure S2. Cluster analysis of cultivation-dependent (A) and cultivation-independent (B) from rape based on DGGE profiles.

Table S1. Assignment of taxonomic groups to band sequences extracted from a DGGE gel and the closest sequence match of known phylogenetic affiliation.

Figure S1


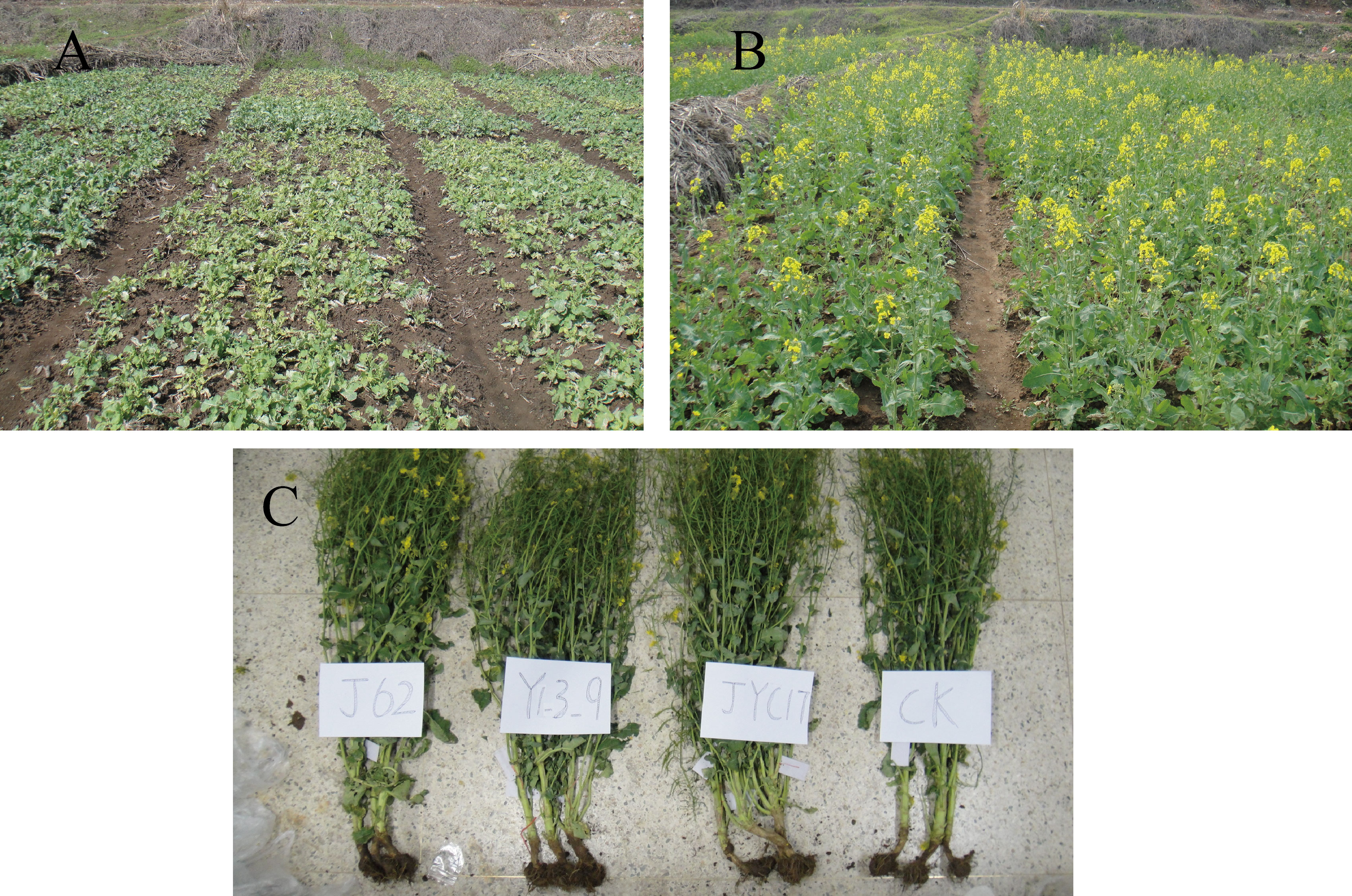


Figure S2


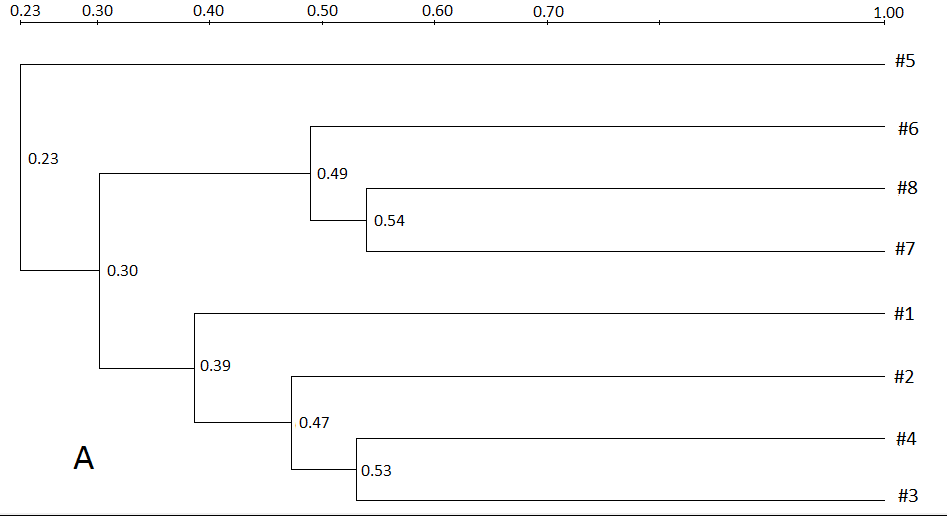


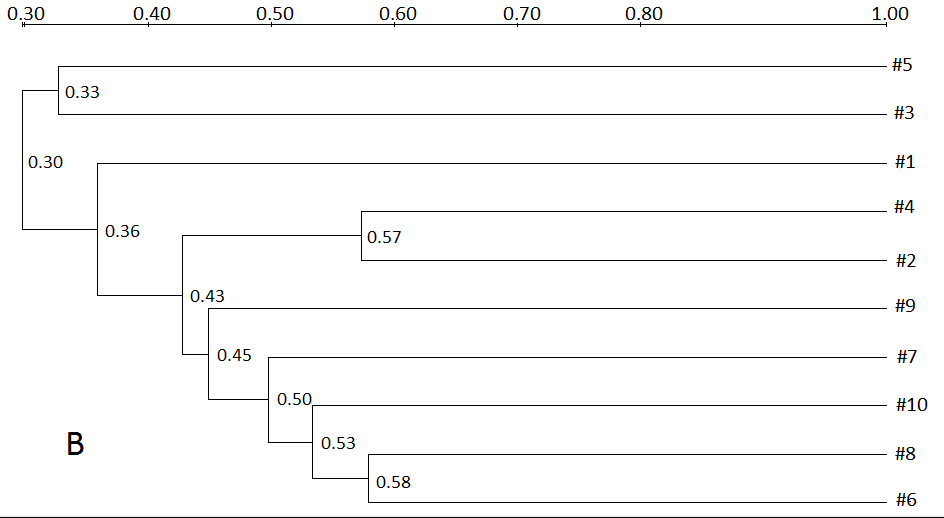


Table S1

| Band | Identity (GenBank no) | Taxonomic group | Similarity |
| --- | --- | --- | --- |
| A-1 (HQ603005) | *Pseudomonas fluorescens* strain HXQ-N33 (HM439651) | Gammaproteobacteria | 100% |
| A-2 (HQ603006) | *Pseudomonas* sp. Y3-3 (HM057106) | Gammaproteobacteria | 100% |
| A-3 (HQ603007) | *Pseudomonas* sp. Y3-3 (HM057106) | Gammaproteobacteria | 100% |
| A-4 (HQ603008) | *Pseudomonas putida* strain NBAII OTN-5 E-2 (HM439974) | Gammaproteobacteria | 100% |
| A-5 (HQ603009) | *Bacillus pumilus* strain BS87 (GQ254767) | Firmicutes | 98% |
| A-6 (HQ603010) | *Pseudomonas fluorescens* strain WR0712 (HM438961) | Gammaproteobacteria | 100% |
| A-7 (HQ603011) | *Bacillus megaterium* strain WIF10 (HM480307) | Firmicutes | 97% |
| A-8 (HQ603012) | *Pseudomonas fluorescens* strain WR0712 (HM438961) | Gammaproteobacteria | 100% |
| A-9 (HQ603013) | *Pseudomonas fluorescens* strain WR0712 (HM438961) | Gammaproteobacteria | 100% |
| A-10 (HQ603014) | *Pseudomonas putida* strain NBAII RPF-9 (HM439967) | Gammaproteobacteria | 100% |
| A-11 (HQ603015) | *Zoogloea* sp. VPMK12 (FM999992) | Betaproteobacteria | 98% |
| A-14 (HQ603016) | *Bacillus aryabhattai* strain OFB-3 (HM439455) | Firmicutes | 100% |
| **A-15 (HQ603017)** | ***Pseudomonas* sp. Y1-3-9 (EU781541)** | **Gammaproteobacteria** | **100%** |
| **A-16 (HQ603018)** | ***Burkholderia* sp*.* J62 (EF555575)** | **Betaproteobacteria** | **100%** |
| A-17 (HQ603019) | *Bacillus pumilus* strain JMC-UBL 20 (HM451441) | Firmicutes | 99% |
| A-18 (HQ603020) | *Bacillus subtilis* strain MA4 (HQ263251) | Firmicutes | 100% |
| A-19 (HQ603021) | *Achromobacter xylosoxidans* strain X96 (HM137034) | Betaproteobacteria | 100% |
| A-20 (HQ603022) | *Pseudomonas fluorescens* strain WR0712 (HM438961) | Gammaproteobacteria | 100% |
| A-21 (HQ603023) | *Bacillus megaterium* strain AK39883 (HQ234352) | Firmicutes | 100% |
| A-22 (HQ603024) | *Paenibacillus* sp. G-41-1 (HM044382) | Firmicutes | 94% |
| **A-23 (HQ603025)** | ***Microbacterium* sp. JYC17 (EU036699)** | **Actinobacteria** | **100%** |
| B-2 (HQ603026) | *Pseudomonas koreensis* strain M9c (GU078448) | Gammaproteobacteria | 100% |
| B-4 (HQ603027) | Uncultured bacterium clone Bac_SH28 (HM559795) | Uncultured | 100% |
| B-6 (HQ603028) | Uncultured bacterium clone J005-I19 (HM988763) | Uncultured | 100% |
| B-7 (HQ603029) | *Burkholderia cepacia* strain 8201 (FJ8705549) | Betaproteobacteria | 100% |
| B-8 (HQ603030) | Uncultured bacterium clone 2-32 (DQ278825) | Uncultured | 91% |
| B-9 (HQ603031) | Mucilaginibacter sp. HME6827 (HQ167732) | Bacteroidetes | 98% |
| B-10 (HQ603032) | *Pedobacter* sp. SANK 72003 (AB522429) | Bacteroidetes | 100% |
| B-14 (HQ603033) | *Pseudomonas putida* strain XJ-2 (HM641753) | Gammaproteobacteria | 99% |
| B-16 (HQ603034) | *Janthinobacterium* sp. 286 (GU213421) | Betaproteobacteria | 98% |
| B-17 (HQ603035) | *Bacillus megaterium* strain NBAII-65 (HQ162496) | Firmicutes | 99% |
| B-18 (HQ603036) | *Rhodanobacter* sp. V3M40 (FN794233) | Gammaproteobacteria | 100% |
| B-19 (HQ603037) | Uncultured bacterium clone nbu139f08c1 (GQ018496) | Uncultured | 97% |
| B-21 (HQ603038) | *Rhodanobacter* sp. T2-YC6779 (GQ369047) | Gammaproteobacteria | 100% |
| B-22 (HQ603039) | *Bacillus pumilus* strain AK39885 (HQ234353) | Firmicutes | 100% |
| B-23 (HQ603040) | *Rhodanobacter* sp. T2-YC6779 (GQ369047) | Gammaproteobacteria | 99% |
| B-24 (HQ603041) | *Phenylobacterium* sp. T2-YC6789 (GQ369057) | Alphaproteobacteria | 100% |
| B-25 (HQ603042) | Uncultured bacterium clone 154 (HM241019) | Uncultured | 92% |
| B-26 (HQ603043) | Uncultured bacterium clone SD8 (AY591523) | Uncultured | 100% |
| B-28 (HQ603044) | Uncultured soil bacterium clone S098 (AY037618) | Uncultured | 100% |
| B-30 (HQ603045) | *Frigoribacterium* sp. YUST-DW3 (HM640282) | Actinobacteria | 100% |
| B-31 (HQ603046) | Uncultured *Chloroflexi* bacterium clone RUGL6-82 (GQ366438) | Uncultured | 100% |
| B-34 (HQ603047) | Uncultured *Halomonas* sp. clone 292 (GU556504) | Uncultured | 100% |
| B-38 (HQ603048) | *Arthrobacter oryzae* strain NBGD40 (HQ003445) | Actinobacteria | 100% |
| B-40 (HQ603049) | *Pedobacter* sp. SANK 72003 (AB5224299) | Bacteroidetes | 100% |
| B-42 (HQ603050) | Uncultured bacterium clone G0-20 (HQ132120) | Uncultured | 99% |
| B-43 (HQ603051) | Uncultured bacterium clone AN103 (GQ859990) | Uncultured | 100% |
